# Snare-tipped endoscopic radical incision and cutting for postoperative colorectal anastomotic stricture

**DOI:** 10.1055/a-2186-5286

**Published:** 2023-11-20

**Authors:** Chung-Ying Lee, Tung-Cheng Chang, Ming-Yao Chen

**Affiliations:** 1 499996 Division of Gastroenterology and Hepatology, Department of Internal Medicine, Taipei Medical University Shuang Ho Hospital Ministry of Health and Welfare, New Taipei City, Taiwan; 2 210822 Gastroenterology and Hepatology, Taipei Medical University School of Medicine, Taipei, Taiwan; 3 38032 TMU Research Center for Digestive Medicine, Taipei Medical University, Taipei, Taiwan; 4 499996 Division of Colorectal Surgery, Department of Surgery, Taipei Medical University Shuang Ho Hospital Ministry of Health and Welfare, New Taipei City, Taiwan; 5 210822 Surgery, Taipei Medical University School of Medicine, Taipei, Taiwan

Postoperative colorectal anastomotic strictures are not uncommon and often require endoscopic or surgical intervention 1 . Initial treatment typically involves endoscopic balloon dilation, which may need to be repeated for long-term efficacy 2 3 . Endoscopic radical incision and cutting has emerged as a novel technique for the treatment of refractory strictures. This technique enables the direct removal of fibrotic scar tissue, reducing the risk of restenosis 2 4 5 . Herein, we introduce a safe and cost-effective method of performing radical incision and cutting using a snare tip to treat benign anastomotic strictures.

A 59-year-old man underwent anterior resection and loop colostomy for sigmoid colon diverticulitis with perforation. A follow-up colonoscopy within 3 months revealed the formation of circular scars and stricturing at the anastomotic site ( Fig. 1 ). The standard colonoscope (distal end/outer diameter 12.2/13.7 mm; CF-Q260AI; Olympus, Japan) could not be passed through the stricture. The patient was referred to us for endoscopic management before the closure of the temporary colostomy.

**Fig. 1:**
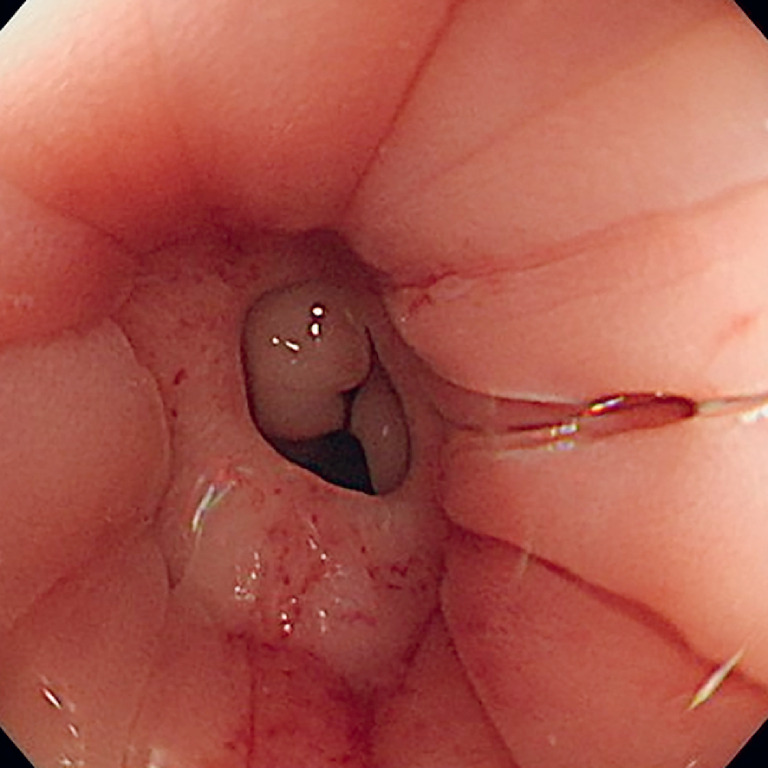
Endoscopic image showing circular scar formation that has resulted in luminal stricture at the anastomotic site.

Instead of endoscopic balloon dilation, we employed an innovative technique called snare-tipped endoscopic radical incision and cutting (STERIC) to address the postoperative anastomotic stricture. We used the tip of the snare (25-mm Snare Master; SD-210U-25; Olympus), sticking out by 2 mm in length, to perform the radical incision and cutting. The snare tip was placed at the edge of the stricture ring at the 6-oʼclock position and then used to make a circumferential incision with the assistance of the electrosurgical unit (VIO 3; Erbe) in Endocut Q mode (effect 2, duration 2, interval 4), employing a step-by-step excision along the arc of the lumen ( Fig. 2 ; Video 1 ). Following complete removal of the circular scar, it was possible to pass the standard colonoscope freely through the anastomotic site, achieving clinical success in only one session.

**Fig. 2:**
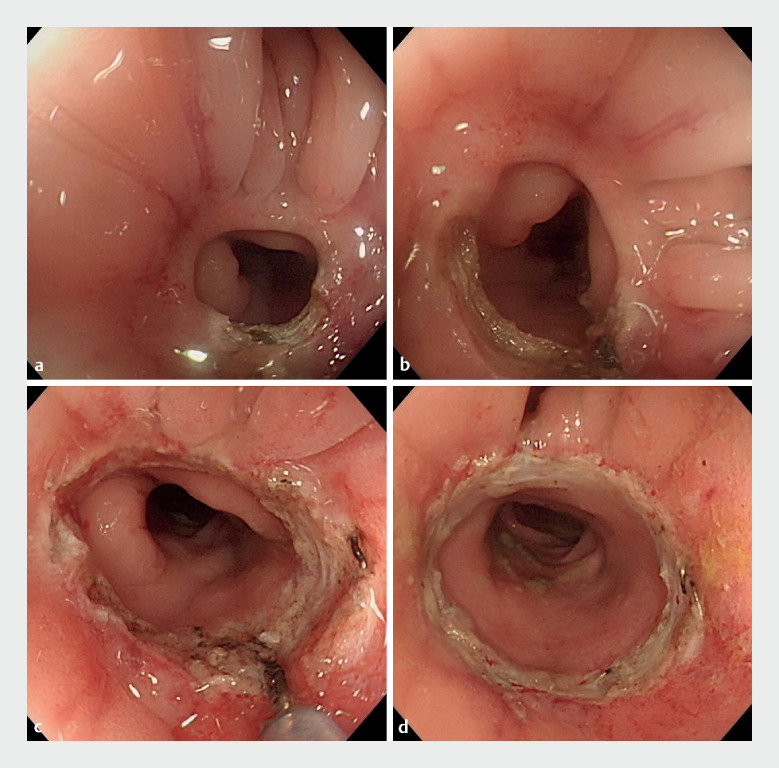
Endoscopic images showing progressive radical incision and cutting along the circular scar in a circumferential manner using a snare tip.

Snare-tipped endoscopic radical incision and cutting (STERIC) is performed for a postoperative colorectal anastomotic stricture.Video 1

The patient subsequently underwent loop transverse colostomy closure, with no signs of restenosis during follow-up.

Endoscopy_UCTN_Code_TTT_1AQ_2AF

## Notes

Endoscopy E-Videos https://eref.thieme.de/e-videos
*E-Videos* is an open access online section of the journal *Endoscopy* , reporting on interesting cases and new techniques in gastroenterological endoscopy. All papers include a high-quality video and are published with a Creative Commons CC-BY license. Endoscopy E-Videos qualify for HINARI discounts and waivers and eligibility is automatically checked during the submission process. We grant 100% waivers to articles whose corresponding authors are based in Group A countries and 50% waivers to those who are based in Group B countries as classified by Research4Life (see: https://www.research4life.org/access/eligibility/ ). This section has its own submission website at https://mc.manuscriptcentral.com/e-videos .
